# 5,11,17,23-Tetra-*tert*-butyl-25,26,27,28-tetra­methoxy­calix[4]arene dichloro­methane hemisolvate

**DOI:** 10.1107/S1600536808002304

**Published:** 2008-03-05

**Authors:** Conrad Fischer, Tobias Gruber, Wilhelm Seichter, Diana Schindler, Edwin Weber

**Affiliations:** a Institut für Organische Chemie, TU Bergakademie Freiberg, Leipziger Strasse 29, D-09596 Freiberg/Sachsen, Germany

## Abstract

In the title compound, C_48_H_64_O_4_·0.5CH_2_Cl_2_, both crystallographically independent calixarene mol­ecules display a partial cone conformation. Their crystal packing is stabilized by C—H⋯π contacts involving the meth­oxy groups. The solvent mol­ecule is located inter­stitially between two calixarene units with C—H⋯Cl contacts to meth­oxy and *tert*-butyl groups. One *tert*-butyl residue of each calixarene mol­ecule is disordered over two positions (occupancies 0.60/0.40 and 0.63/0.37), resulting in bond distances that deviate from ideal values. The tetra­mer calixarene mol­ecules present models with approximate non-crystallographic *C_s_* symmetry.

## Related literature

The solvent-free title compound has been described to assume a *partial cone* conformation (Grootenhuis *et al.*, 1990 ▶). A closely related solvate with THF has been described previously (Fischer *et al.*, 2007 ▶, and literature cited therein). For the synthesis of the compound, see: Bitter *et al.* (1995 ▶). For other related literature, see: Gutsche *et al.* (1983 ▶), Iwamoto *et al.* (1991 ▶); Nishio (2004 ▶).
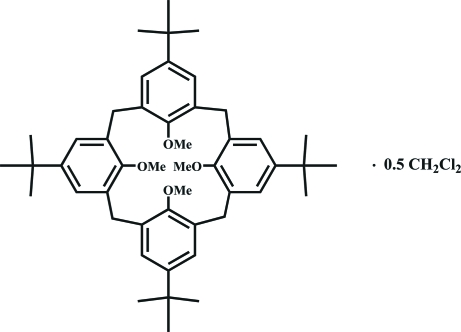


## Experimental

### 

#### Crystal data

C_48_H_64_O_4_·0.5CH_2_Cl_2_
*M* *_r_* = 747.46
Monoclinic, 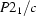

*a* = 16.7012 (6) Å
*b* = 19.7113 (8) Å
*c* = 28.2577 (11) Å
β = 103.097 (2)°
*V* = 9060.5 (6) Å^3^
*Z* = 8
Mo *K*α radiation
μ = 0.12 mm^−1^
*T* = 133 (2) K
0.60 × 0.60 × 0.60 mm

#### Data collection

Bruker Kappa APEXII CCD diffractometer
Absorption correction: multi-scan (*SADABS*; Sheldrick, 2004 ▶) *T*_min_ = 0.929, *T*_max_ = 0.929
186561 measured reflections
20781 independent reflections
12758 reflections with *I* > 2σ(*I*)
*R*_int_ = 0.051

#### Refinement

*R*[*F* ^2^ > 2σ(*F* ^2^)] = 0.073
*wR*(*F* ^2^) = 0.272
*S* = 1.21
20781 reflections
1058 parameters
H-atom parameters constrained
Δρ_max_ = 0.82 e Å^−3^
Δρ_min_ = −1.13 e Å^−3^

### 

Data collection: *APEX2* (Bruker, 2004 ▶); cell refinement: *SAINT-NT* (Bruker, 2004 ▶); data reduction: *SAINT-NT*; program(s) used to solve structure: *SHELXS97* (Sheldrick, 2008 ▶); program(s) used to refine structure: *SHELXL97* (Sheldrick, 2008 ▶); molecular graphics: *SHELXTL* (Sheldrick, 2008 ▶); software used to prepare material for publication: *SHELXTL*.

## Supplementary Material

Crystal structure: contains datablocks global, I. DOI: 10.1107/S1600536808002304/si2068sup1.cif

Structure factors: contains datablocks I. DOI: 10.1107/S1600536808002304/si2068Isup2.hkl

Additional supplementary materials:  crystallographic information; 3D view; checkCIF report

## Figures and Tables

**Table 1:** Hydrogen-bond geometry (Å, °)

*D*—H⋯*A*	*D*—H	H⋯*A*	*D*⋯*A*	*D*—H⋯*A*
C33*A*—H33*E*⋯Cl1*G*^i^	0.98	2.92	3.711 (3)	139
C46—H46*A*⋯Cl2*G*^ii^	0.98	2.89	3.551 (10)	126
C7*A*—H7*A*1⋯*CgC*^i^	0.99	2.88	3.733 (2)	145
C33—H33*A*⋯*CgA′*^iii^	0.98	2.74	3.549 (3)	140
C48—H48*A*⋯*CgC′*	0.98	2.76	3.526 (3)	136

Symmetry codes: (i) 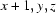; (ii) 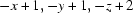; (iii) 
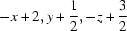
. *CgC* is the centroid of atoms C15–C20, *CgA*′ is the centroid of atoms C1*A*–C6*A *and *CgC*′ is the centroid of atoms C15*A*–C20*A.*

**Fig. 1.**
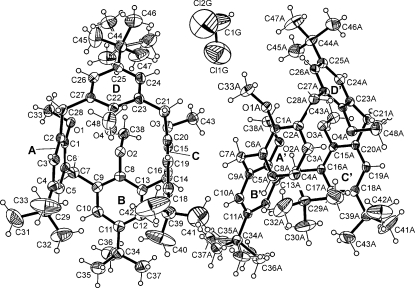
Molecular presentation of the title compound with CH2Cl2 as guest.

**Fig. 2.**
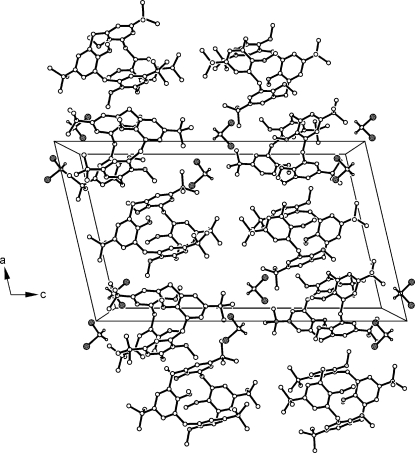
Packing diagram, viewed down the b axis. H atoms have been omitted for clarity.

## supplementary crystallographic information

### Comment

The molecular geometry of the calixarene (Figure 1) is best described by a *partial cone* conformation with opposite arene rings A and C differing only little from coplanarity [5.6 (1) and 9.2 (1)°, respectively], whereas the aromatic rings B and D include dihedral angles of 54.9 (1) and 58.4 (1)°, respectively. The solvent molecules are everything but located inside of the calixarene cavity offering not enough space for an accommodation. Hence the CH_2_Cl_2_ molecule occupies interstitial lattice space between two calixarene molecules (Figure 2). Due to their hydrophobic surface, the packing of the calixarene molecules is primary stabilized by van-der-Waals interactions and weak C—H···π contacts (Nishio, 2004) involving methoxy groups and neighboring aromatic rings, with H···π (centroid of the aromatic ring) distances ranging from 2.64 to 2.93 Å. Furthermore, weak C—H···Cl interactions including one of the methoxy groups [*d*(C33A···Cl1G) = 3.6554 Å] and a *tert*-butyl group [*d*(C46···Cl2G) = 3.4833 Å] can be observed (Table 1). The *tert*-butyl residues are partially disordered over two positions [0.60/0.40 (ring D), 0.63/0.37 (ring B')], resulting in bond distances that deviate from ideal values.

### Experimental

The title calixarene was sythesized from 5,11,17,23-tetra-*tert*-butyl-25,26,27,28-tetrahydroxycalix[4]arene following the literature procedure (Bitter *et al.*, 1995). Colourless prisms of the solvated calixarene suitable for X-ray diffraction were obtained by recrystallization from dichloromethane (m.p. 515–517 K).

### Refinement

The H atoms were positioned geometrically and allowed to ride on their parent atoms, with C—H = 0.95 Å, and *U*_iso_(H) = 1.2–1.5 times *U*_eq_ (parent atom).

### Crystal data

**Table:**

C_48_H_64_O_4_·0.5CH_2_Cl_2_	*Z* = 8
*M**_r_* = 747.46	*F*_000_ = 3240
Monoclinic, *P*2_1_/*c*	*D*_x_ = 1.096 Mg m^−^^3^
Hall symbol: -P 2ybc	Mo *K*α radiation λ = 0.71073 Å
*a* = 16.7012 (6) Å	µ = 0.12 mm^−^^1^
*b* = 19.7113 (8) Å	*T* = 133 (2) K
*c* = 28.2577 (11) Å	Irregular, colourless
β = 103.097 (2)º	0.60 × 0.60 × 0.60 mm
*V* = 9060.5 (6) Å^3^	

### Data collection

**Table:**

Bruker Kappa APEXII CCD diffractometer	20781 independent reflections
Radiation source: fine-focus sealed tube	12758 reflections with *I* > 2σ(*I*)
Monochromator: graphite	*R*_int_ = 0.051
*T* = 133(2) K	θ_max_ = 27.5º
φ and ω scans	θ_min_ = 1.3º
Absorption correction: multi-scan(SADABS; Sheldrick, 2004)	*h* = −21→19
*T*_min_ = 0.929, *T*_max_ = 0.929	*k* = −25→25
186561 measured reflections	*l* = −36→36

### Refinement

**Table:**

Refinement on *F*^2^	Secondary atom site location: difference Fourier map
Least-squares matrix: full	Hydrogen site location: inferred from neighbouring sites
*R*[*F*^2^ > 2σ(*F*^2^)] = 0.073	H-atom parameters constrained
*wR*(*F*^2^) = 0.272	*w* = 1/[σ^2^(*F*_o_^2^) + (0.1495*P*)^2^ + 1.8277*P*] where *P* = (*F*_o_^2^ + 2*F*_c_^2^)/3
*S* = 1.21	(Δ/σ)_max_ = 0.040
20781 reflections	Δρ_max_ = 0.83 e Å^−^^3^
1058 parameters	Δρ_min_ = −1.13 e Å^−^^3^
Primary atom site location: structure-invariant direct methods	Extinction correction: none

### Special details

**Table:**

Geometry. All e.s.d.'s (except the e.s.d. in the dihedral angle between two l.s. planes) are estimated using the full covariance matrix. The cell e.s.d.'s are taken into account individually in the estimation of e.s.d.'s in distances, angles and torsion angles; correlations between e.s.d.'s in cell parameters are only used when they are defined by crystal symmetry. An approximate (isotropic) treatment of cell e.s.d.'s is used for estimating e.s.d.'s involving l.s. planes.
Refinement. Refinement of *F*^2^ against ALL reflections. The weighted *R*-factor *wR* and goodness of fit *S* are based on *F*^2^, conventional *R*-factors *R* are based on *F*, with *F* set to zero for negative *F*^2^. The threshold expression of *F*^2^ > σ(*F*^2^) is used only for calculating *R*-factors(gt) *etc*. and is not relevant to the choice of reflections for refinement. *R*-factors based on *F*^2^ are statistically about twice as large as those based on *F*, and *R*-factors based on ALL data will be even larger.

### Fractional atomic coordinates and isotropic or equivalent isotropic displacement parameters (Å^2^)

**Table:**

	*x*	*y*	*z*	*U*_iso_*/*U*_eq_	Occ. (<1)
O1	0.60280 (10)	0.76526 (9)	0.76127 (6)	0.0273 (4)	
O2	0.42283 (10)	0.73542 (8)	0.71821 (6)	0.0265 (4)	
O3	0.34908 (10)	0.62305 (9)	0.76518 (6)	0.0280 (4)	
O4	0.57295 (11)	0.54128 (9)	0.72312 (6)	0.0311 (4)	
C1	0.60986 (14)	0.74154 (12)	0.71608 (9)	0.0238 (5)	
C2	0.65502 (14)	0.68252 (13)	0.71359 (9)	0.0252 (5)	
C3	0.66089 (15)	0.65863 (14)	0.66799 (9)	0.0293 (5)	
H3	0.6913	0.6184	0.6660	0.035*	
C4	0.62337 (16)	0.69217 (14)	0.62514 (9)	0.0302 (6)	
C5	0.57696 (16)	0.74944 (13)	0.62940 (9)	0.0296 (6)	
H5	0.5500	0.7726	0.6007	0.035*	
C6	0.56842 (15)	0.77421 (12)	0.67407 (9)	0.0252 (5)	
C7	0.51028 (16)	0.83291 (13)	0.67603 (10)	0.0302 (6)	
H7A	0.5139	0.8460	0.7103	0.036*	
H7B	0.5264	0.8726	0.6588	0.036*	
C8	0.38253 (15)	0.76378 (12)	0.67482 (9)	0.0243 (5)	
C9	0.42270 (15)	0.81254 (12)	0.65258 (9)	0.0267 (5)	
C10	0.38306 (16)	0.83698 (13)	0.60731 (10)	0.0316 (6)	
H10	0.4094	0.8710	0.5924	0.038*	
C11	0.30530 (16)	0.81335 (13)	0.58257 (10)	0.0309 (6)	
C12	0.26764 (15)	0.76518 (13)	0.60595 (10)	0.0296 (6)	
H12	0.2147	0.7489	0.5902	0.035*	
C13	0.30470 (15)	0.73966 (12)	0.65185 (9)	0.0257 (5)	
C14	0.26261 (15)	0.68638 (13)	0.67634 (10)	0.0287 (5)	
H14C	0.2046	0.6820	0.6585	0.034*	
H14D	0.2631	0.7011	0.7099	0.034*	
C15	0.34700 (14)	0.58927 (11)	0.72173 (9)	0.0232 (5)	
C16	0.30485 (14)	0.61746 (12)	0.67787 (9)	0.0236 (5)	
C17	0.30437 (15)	0.58322 (13)	0.63478 (9)	0.0278 (5)	
H17	0.2757	0.6025	0.6049	0.033*	
C18	0.34481 (16)	0.52136 (13)	0.63419 (10)	0.0304 (6)	
C19	0.38780 (16)	0.49575 (12)	0.67860 (10)	0.0284 (5)	
H19	0.4162	0.4539	0.6790	0.034*	
C20	0.39083 (14)	0.52889 (12)	0.72225 (9)	0.0236 (5)	
C21	0.44439 (16)	0.50240 (13)	0.76944 (10)	0.0293 (6)	
H21C	0.4094	0.4937	0.7928	0.035*	
H21D	0.4691	0.4587	0.7630	0.035*	
C22	0.57354 (14)	0.56945 (12)	0.76836 (9)	0.0243 (5)	
C23	0.51265 (14)	0.55093 (12)	0.79241 (9)	0.0254 (5)	
C24	0.51367 (16)	0.58064 (14)	0.83718 (10)	0.0302 (6)	
H24	0.4727	0.5680	0.8540	0.036*	
C25	0.57315 (16)	0.62863 (14)	0.85844 (9)	0.0316 (6)	
C26	0.63137 (16)	0.64655 (14)	0.83222 (9)	0.0293 (5)	
H26	0.6717	0.6796	0.8455	0.035*	
C27	0.63274 (14)	0.61798 (12)	0.78739 (9)	0.0242 (5)	
C28	0.69414 (15)	0.64219 (14)	0.75891 (9)	0.0274 (5)	
H28C	0.7228	0.6023	0.7492	0.033*	
H28D	0.7359	0.6709	0.7803	0.033*	
C29	0.6327 (2)	0.66758 (17)	0.57503 (10)	0.0466 (8)	
C30	0.6708 (4)	0.5982 (3)	0.57684 (15)	0.1008 (19)	
H30A	0.7255	0.5990	0.5987	0.151*	
H30B	0.6757	0.5850	0.5442	0.151*	
H30C	0.6361	0.5653	0.5888	0.151*	
C31	0.6929 (4)	0.7179 (3)	0.55764 (17)	0.1027 (19)	
H31A	0.6734	0.7645	0.5593	0.154*	
H31B	0.6950	0.7073	0.5241	0.154*	
H31C	0.7481	0.7134	0.5786	0.154*	
C32	0.5536 (3)	0.6726 (3)	0.53776 (14)	0.0903 (16)	
H32A	0.5128	0.6424	0.5468	0.135*	
H32B	0.5625	0.6591	0.5060	0.135*	
H32C	0.5335	0.7194	0.5361	0.135*	
C33	0.66670 (17)	0.81078 (14)	0.78376 (10)	0.0335 (6)	
H33A	0.7202	0.7913	0.7825	0.050*	
H33B	0.6642	0.8180	0.8177	0.050*	
H33C	0.6596	0.8543	0.7665	0.050*	
C34	0.26726 (19)	0.84015 (16)	0.53167 (11)	0.0418 (7)	
C35	0.3259 (2)	0.8256 (2)	0.49815 (12)	0.0536 (9)	
H35A	0.3778	0.8497	0.5101	0.080*	
H35B	0.3008	0.8409	0.4651	0.080*	
H35C	0.3365	0.7767	0.4978	0.080*	
C36	0.2521 (2)	0.91672 (18)	0.53399 (14)	0.0592 (10)	
H36A	0.2099	0.9251	0.5524	0.089*	
H36B	0.2335	0.9346	0.5010	0.089*	
H36C	0.3033	0.9394	0.5501	0.089*	
C37	0.1848 (2)	0.8052 (2)	0.50887 (13)	0.0614 (10)	
H37A	0.1934	0.7561	0.5073	0.092*	
H37B	0.1636	0.8229	0.4760	0.092*	
H37C	0.1451	0.8144	0.5288	0.092*	
C38	0.41349 (18)	0.77014 (15)	0.76099 (10)	0.0354 (6)	
H38D	0.4361	0.8161	0.7613	0.053*	
H38E	0.4430	0.7454	0.7898	0.053*	
H38F	0.3550	0.7728	0.7613	0.053*	
C39	0.3431 (2)	0.48364 (16)	0.58658 (11)	0.0442 (7)	
C40	0.3160 (5)	0.5286 (3)	0.54277 (16)	0.123 (3)	
H40A	0.3502	0.5695	0.5466	0.185*	
H40B	0.2584	0.5414	0.5396	0.185*	
H40C	0.3219	0.5040	0.5136	0.185*	
C41	0.2830 (5)	0.4240 (3)	0.58312 (18)	0.119 (2)	
H41A	0.2978	0.3886	0.5623	0.179*	
H41B	0.2270	0.4399	0.5692	0.179*	
H41C	0.2859	0.4055	0.6156	0.179*	
C42	0.4238 (5)	0.4509 (5)	0.5877 (2)	0.188 (5)	
H42A	0.4447	0.4300	0.6196	0.282*	
H42B	0.4630	0.4851	0.5818	0.282*	
H42C	0.4169	0.4159	0.5625	0.282*	
C43	0.28403 (18)	0.60501 (15)	0.78743 (11)	0.0371 (6)	
H43A	0.2313	0.6117	0.7643	0.056*	
H43B	0.2862	0.6336	0.8160	0.056*	
H43C	0.2897	0.5573	0.7973	0.056*	
C44	0.57290 (18)	0.66186 (17)	0.90742 (10)	0.0406 (7)	
C45B	0.5733 (5)	0.7411 (3)	0.9013 (2)	0.063 (2)	0.599 (9)
H45D	0.5230	0.7554	0.8783	0.094*	0.599 (9)
H45E	0.6212	0.7546	0.8889	0.094*	0.599 (9)
H45F	0.5760	0.7628	0.9328	0.094*	0.599 (9)
C46B	0.6508 (4)	0.6418 (4)	0.94219 (19)	0.056 (2)	0.599 (9)
H46D	0.6535	0.6638	0.9736	0.085*	0.599 (9)
H46E	0.6979	0.6561	0.9293	0.085*	0.599 (9)
H46F	0.6520	0.5925	0.9464	0.085*	0.599 (9)
C47B	0.4999 (5)	0.6429 (6)	0.9272 (3)	0.086 (4)	0.599 (9)
H47D	0.5012	0.5940	0.9339	0.129*	0.599 (9)
H47E	0.4493	0.6542	0.9034	0.129*	0.599 (9)
H47F	0.5016	0.6679	0.9574	0.129*	0.599 (9)
C48	0.61588 (19)	0.47793 (16)	0.72585 (13)	0.0491 (8)	
H48A	0.6751	0.4859	0.7369	0.074*	
H48B	0.6045	0.4567	0.6937	0.074*	
H48C	0.5974	0.4479	0.7488	0.074*	
C45	0.6427 (9)	0.7115 (7)	0.9256 (4)	0.086 (5)	0.401 (9)
H45A	0.6489	0.7190	0.9605	0.129*	0.401 (9)
H45B	0.6304	0.7547	0.9083	0.129*	0.401 (9)
H45C	0.6939	0.6929	0.9196	0.129*	0.401 (9)
C46	0.5904 (8)	0.6036 (7)	0.9475 (3)	0.080 (4)	0.401 (9)
H46A	0.5985	0.6239	0.9799	0.121*	0.401 (9)
H46B	0.6399	0.5785	0.9449	0.121*	0.401 (9)
H46C	0.5434	0.5725	0.9423	0.121*	0.401 (9)
C47	0.4894 (8)	0.6916 (8)	0.9057 (4)	0.080 (5)	0.401 (9)
H47A	0.4473	0.6569	0.8949	0.121*	0.401 (9)
H47B	0.4798	0.7297	0.8828	0.121*	0.401 (9)
H47C	0.4867	0.7077	0.9381	0.121*	0.401 (9)
O1A	1.17566 (10)	0.39965 (9)	0.79774 (6)	0.0261 (4)	
O2A	1.03065 (10)	0.48985 (8)	0.75598 (6)	0.0253 (4)	
O3A	0.88555 (10)	0.49010 (8)	0.80712 (6)	0.0271 (4)	
O4A	0.94633 (10)	0.27854 (8)	0.76393 (6)	0.0263 (4)	
C1A	1.14831 (13)	0.36509 (12)	0.75435 (8)	0.0222 (5)	
C2A	1.12314 (14)	0.29761 (12)	0.75532 (9)	0.0238 (5)	
C3A	1.09746 (15)	0.26289 (12)	0.71166 (9)	0.0251 (5)	
H3A	1.0822	0.2165	0.7123	0.030*	
C4A	1.09350 (14)	0.29436 (13)	0.66678 (9)	0.0256 (5)	
C5A	1.11584 (15)	0.36271 (13)	0.66732 (9)	0.0261 (5)	
H5A	1.1125	0.3854	0.6373	0.031*	
C6A	1.14280 (14)	0.39868 (12)	0.71022 (9)	0.0236 (5)	
C7A	1.16345 (15)	0.47365 (12)	0.70879 (10)	0.0271 (5)	
H7A1	1.2047	0.4799	0.6890	0.032*	
H7A2	1.1881	0.4894	0.7422	0.032*	
C8A	1.02534 (15)	0.52380 (11)	0.71257 (9)	0.0231 (5)	
C9A	1.08823 (15)	0.51668 (12)	0.68767 (9)	0.0253 (5)	
C10A	1.07839 (16)	0.54748 (12)	0.64226 (9)	0.0271 (5)	
H10A	1.1213	0.5435	0.6254	0.033*	
C11A	1.00809 (16)	0.58383 (12)	0.62079 (9)	0.0281 (5)	
C12A	0.94694 (16)	0.58977 (12)	0.64691 (9)	0.0274 (5)	
H12A	0.8987	0.6147	0.6330	0.033*	
C13A	0.95378 (15)	0.56060 (11)	0.69247 (9)	0.0246 (5)	
C14A	0.88412 (15)	0.56362 (12)	0.71865 (9)	0.0258 (5)	
H14A	0.8421	0.5963	0.7019	0.031*	
H14B	0.9054	0.5799	0.7523	0.031*	
C15A	0.84881 (13)	0.45960 (12)	0.76320 (9)	0.0214 (5)	
C16A	0.84482 (14)	0.49415 (12)	0.71992 (9)	0.0226 (5)	
C17A	0.80957 (15)	0.46159 (12)	0.67593 (9)	0.0258 (5)	
H17A	0.8058	0.4855	0.6463	0.031*	
C18A	0.78017 (14)	0.39570 (12)	0.67430 (9)	0.0245 (5)	
C19A	0.78754 (14)	0.36195 (12)	0.71858 (9)	0.0243 (5)	
H19A	0.7680	0.3167	0.7183	0.029*	
C20A	0.82237 (14)	0.39208 (12)	0.76307 (9)	0.0226 (5)	
C21A	0.83696 (14)	0.35087 (12)	0.80954 (9)	0.0246 (5)	
H21A	0.8117	0.3055	0.8022	0.030*	
H21B	0.8093	0.3735	0.8328	0.030*	
C22A	0.97906 (14)	0.30570 (11)	0.80969 (8)	0.0219 (5)	
C23A	0.92743 (14)	0.34220 (11)	0.83324 (9)	0.0220 (5)	
C24A	0.96146 (15)	0.37132 (12)	0.87778 (9)	0.0254 (5)	
H24A	0.9264	0.3952	0.8942	0.031*	
C25A	1.04513 (15)	0.36707 (12)	0.89966 (9)	0.0256 (5)	
C26A	1.09458 (15)	0.33049 (12)	0.87496 (9)	0.0256 (5)	
H26A	1.1516	0.3262	0.8892	0.031*	
C27A	1.06315 (15)	0.30008 (12)	0.83016 (8)	0.0235 (5)	
C28A	1.11987 (15)	0.26372 (12)	0.80311 (9)	0.0251 (5)	
H28A	1.1760	0.2623	0.8241	0.030*	
H28B	1.1008	0.2164	0.7966	0.030*	
C29A	1.06832 (16)	0.25501 (14)	0.61851 (9)	0.0324 (6)	
C30A	1.0112 (3)	0.2970 (2)	0.58006 (13)	0.0701 (12)	
H30D	0.9668	0.3156	0.5936	0.105*	
H30E	0.9879	0.2683	0.5520	0.105*	
H30F	1.0422	0.3343	0.5698	0.105*	
C31A	1.0241 (3)	0.18939 (19)	0.62370 (13)	0.0700 (12)	
H31D	0.9752	0.1991	0.6363	0.105*	
H31E	1.0609	0.1592	0.6463	0.105*	
H31F	1.0074	0.1673	0.5919	0.105*	
C32A	1.1460 (2)	0.2390 (3)	0.60063 (17)	0.103 (2)	
H32D	1.1306	0.2232	0.5669	0.154*	
H32E	1.1774	0.2035	0.6211	0.154*	
H32F	1.1797	0.2800	0.6024	0.154*	
C33A	1.26088 (17)	0.38840 (17)	0.81930 (11)	0.0430 (7)	
H33D	1.2731	0.3398	0.8186	0.064*	
H33E	1.2732	0.4043	0.8530	0.064*	
H33F	1.2947	0.4134	0.8010	0.064*	
C34A	0.99674 (17)	0.61515 (14)	0.56982 (9)	0.0322 (6)	
C35A	0.9877 (4)	0.6895 (2)	0.57200 (19)	0.0547 (17)	0.635 (5)
H35D	0.9740	0.7080	0.5389	0.082*	0.635 (5)
H35E	1.0394	0.7094	0.5900	0.082*	0.635 (5)
H35F	0.9436	0.7004	0.5885	0.082*	0.635 (5)
C36A	0.9233 (3)	0.5804 (3)	0.53682 (17)	0.0476 (14)	0.635 (5)
H36D	0.9160	0.5981	0.5037	0.071*	0.635 (5)
H36E	0.8737	0.5893	0.5488	0.071*	0.635 (5)
H36F	0.9331	0.5313	0.5367	0.071*	0.635 (5)
C37A	1.0737 (3)	0.6005 (3)	0.54710 (16)	0.0412 (12)	0.635 (5)
H37D	1.0828	0.5514	0.5462	0.062*	0.635 (5)
H37E	1.1226	0.6223	0.5671	0.062*	0.635 (5)
H37F	1.0633	0.6188	0.5140	0.062*	0.635 (5)
C38A	1.06639 (17)	0.52777 (14)	0.79879 (10)	0.0324 (6)	
H38A	1.1232	0.5397	0.7984	0.049*	
H38B	1.0659	0.5003	0.8276	0.049*	
H38C	1.0345	0.5693	0.7997	0.049*	
C39A	0.74262 (18)	0.35964 (14)	0.62626 (10)	0.0349 (6)	
C40A	0.7667 (4)	0.3932 (2)	0.58367 (13)	0.0836 (15)	
H40D	0.7493	0.4408	0.5818	0.125*	
H40E	0.7399	0.3697	0.5537	0.125*	
H40F	0.8265	0.3908	0.5878	0.125*	
C41A	0.6505 (3)	0.3582 (4)	0.61861 (19)	0.136 (3)	
H41D	0.6354	0.3370	0.6467	0.204*	
H41E	0.6269	0.3321	0.5893	0.204*	
H41F	0.6292	0.4047	0.6148	0.204*	
C42A	0.7712 (5)	0.2869 (2)	0.62814 (16)	0.128 (3)	
H42D	0.8311	0.2853	0.6393	0.192*	
H42E	0.7550	0.2668	0.5956	0.192*	
H42F	0.7459	0.2613	0.6507	0.192*	
C43A	0.82867 (19)	0.51704 (15)	0.83343 (10)	0.0377 (6)	
H43D	0.8070	0.5604	0.8190	0.057*	
H43E	0.8569	0.5242	0.8674	0.057*	
H43F	0.7833	0.4850	0.8318	0.057*	
C44A	1.07984 (17)	0.40356 (14)	0.94792 (10)	0.0331 (6)	
C45A	1.0699 (2)	0.48036 (17)	0.93963 (13)	0.0556 (9)	
H45G	1.0923	0.5043	0.9702	0.083*	
H45H	1.0115	0.4914	0.9283	0.083*	
H45I	1.0997	0.4945	0.9151	0.083*	
C46A	1.0315 (2)	0.3829 (2)	0.98586 (11)	0.0531 (9)	
H46G	1.0323	0.3334	0.9892	0.080*	
H46H	0.9745	0.3985	0.9753	0.080*	
H46I	1.0568	0.4037	1.0172	0.080*	
C47A	1.1702 (2)	0.3878 (2)	0.96844 (13)	0.0643 (11)	
H47G	1.1778	0.3385	0.9715	0.096*	
H47H	1.1881	0.4089	1.0005	0.096*	
H47I	1.2030	0.4057	0.9465	0.096*	
C48A	0.92307 (18)	0.20899 (14)	0.76376 (11)	0.0360 (6)	
H48D	0.9717	0.1813	0.7769	0.054*	
H48E	0.8987	0.1947	0.7304	0.054*	
H48F	0.8828	0.2032	0.7838	0.054*	
C35C	1.0582 (5)	0.6725 (4)	0.5717 (3)	0.040 (2)	0.365 (5)
H35G	1.1141	0.6540	0.5790	0.060*	0.365 (5)
H35H	1.0525	0.7049	0.5970	0.060*	0.365 (5)
H35I	1.0475	0.6957	0.5402	0.060*	0.365 (5)
C36C	0.9067 (5)	0.6548 (4)	0.5526 (3)	0.038 (2)	0.365 (5)
H36G	0.9020	0.6744	0.5203	0.058*	0.365 (5)
H36H	0.9031	0.6910	0.5759	0.058*	0.365 (5)
H36I	0.8619	0.6223	0.5517	0.058*	0.365 (5)
C37C	0.9978 (7)	0.5637 (5)	0.5339 (3)	0.053 (3)	0.365 (5)
H37G	0.9875	0.5846	0.5016	0.080*	0.365 (5)
H37H	0.9551	0.5300	0.5348	0.080*	0.365 (5)
H37I	1.0517	0.5414	0.5408	0.080*	0.365 (5)
C1G	0.3739 (3)	0.4943 (3)	0.95514 (16)	0.0794 (13)	
H1G1	0.3421	0.4691	0.9750	0.095*	
H1G2	0.4055	0.5302	0.9758	0.095*	
Cl1G	0.30659 (10)	0.53169 (7)	0.90646 (4)	0.0958 (4)	
Cl2G	0.44281 (10)	0.43834 (9)	0.93625 (6)	0.1139 (5)	

### Atomic displacement parameters (Å^2^)

**Table:**

	*U*^11^	*U*^22^	*U*^33^	*U*^12^	*U*^13^	*U*^23^
O1	0.0250 (9)	0.0310 (9)	0.0257 (9)	−0.0065 (7)	0.0055 (7)	−0.0044 (7)
O2	0.0284 (9)	0.0244 (9)	0.0256 (9)	0.0036 (7)	0.0036 (7)	0.0011 (7)
O3	0.0292 (9)	0.0256 (9)	0.0292 (9)	−0.0007 (7)	0.0069 (7)	−0.0024 (7)
O4	0.0289 (9)	0.0338 (10)	0.0308 (10)	0.0044 (8)	0.0071 (8)	−0.0075 (8)
C1	0.0199 (11)	0.0271 (12)	0.0245 (12)	−0.0075 (9)	0.0051 (9)	−0.0024 (10)
C2	0.0200 (11)	0.0301 (13)	0.0248 (12)	−0.0033 (9)	0.0036 (9)	0.0015 (10)
C3	0.0252 (12)	0.0354 (14)	0.0275 (13)	0.0013 (10)	0.0068 (10)	−0.0006 (11)
C4	0.0319 (13)	0.0352 (14)	0.0231 (13)	−0.0027 (11)	0.0054 (10)	−0.0010 (11)
C5	0.0323 (13)	0.0298 (13)	0.0238 (13)	−0.0024 (11)	0.0004 (10)	0.0060 (10)
C6	0.0246 (12)	0.0206 (11)	0.0294 (13)	−0.0064 (9)	0.0043 (10)	−0.0004 (10)
C7	0.0333 (14)	0.0230 (12)	0.0319 (14)	−0.0006 (10)	0.0025 (11)	0.0016 (10)
C8	0.0272 (12)	0.0187 (11)	0.0265 (13)	0.0077 (9)	0.0050 (10)	0.0007 (9)
C9	0.0286 (13)	0.0181 (11)	0.0325 (14)	0.0025 (9)	0.0052 (10)	−0.0015 (10)
C10	0.0342 (14)	0.0241 (13)	0.0368 (15)	0.0044 (10)	0.0088 (11)	0.0053 (11)
C11	0.0297 (13)	0.0319 (14)	0.0304 (14)	0.0100 (11)	0.0051 (11)	0.0040 (11)
C12	0.0247 (12)	0.0304 (13)	0.0317 (14)	0.0069 (10)	0.0026 (10)	−0.0001 (11)
C13	0.0262 (12)	0.0232 (12)	0.0277 (13)	0.0073 (9)	0.0063 (10)	−0.0017 (10)
C14	0.0237 (12)	0.0291 (13)	0.0336 (14)	0.0055 (10)	0.0070 (10)	0.0029 (11)
C15	0.0223 (11)	0.0186 (11)	0.0292 (13)	−0.0036 (9)	0.0070 (10)	−0.0013 (9)
C16	0.0178 (11)	0.0234 (12)	0.0300 (13)	−0.0014 (9)	0.0066 (9)	0.0030 (10)
C17	0.0230 (12)	0.0314 (13)	0.0278 (13)	−0.0025 (10)	0.0030 (10)	0.0030 (10)
C18	0.0319 (14)	0.0296 (13)	0.0309 (14)	−0.0064 (11)	0.0094 (11)	−0.0046 (11)
C19	0.0289 (13)	0.0192 (11)	0.0381 (15)	0.0017 (10)	0.0095 (11)	−0.0003 (10)
C20	0.0203 (11)	0.0199 (11)	0.0299 (13)	−0.0025 (9)	0.0044 (10)	0.0018 (10)
C21	0.0296 (13)	0.0229 (12)	0.0343 (14)	0.0001 (10)	0.0049 (11)	0.0072 (11)
C22	0.0207 (11)	0.0269 (12)	0.0239 (12)	0.0058 (9)	0.0018 (9)	0.0028 (10)
C23	0.0224 (12)	0.0233 (12)	0.0286 (13)	0.0034 (9)	0.0017 (10)	0.0084 (10)
C24	0.0267 (13)	0.0366 (14)	0.0283 (13)	−0.0011 (11)	0.0084 (10)	0.0070 (11)
C25	0.0329 (14)	0.0396 (15)	0.0227 (13)	0.0005 (11)	0.0070 (11)	0.0031 (11)
C26	0.0264 (12)	0.0349 (14)	0.0247 (13)	−0.0002 (11)	0.0021 (10)	0.0042 (11)
C27	0.0190 (11)	0.0289 (13)	0.0238 (12)	0.0048 (9)	0.0032 (9)	0.0046 (10)
C28	0.0201 (11)	0.0359 (14)	0.0253 (13)	0.0025 (10)	0.0034 (10)	0.0010 (10)
C29	0.059 (2)	0.057 (2)	0.0227 (14)	0.0126 (16)	0.0065 (13)	−0.0034 (13)
C30	0.166 (5)	0.093 (3)	0.041 (2)	0.055 (4)	0.018 (3)	−0.014 (2)
C31	0.145 (5)	0.123 (4)	0.062 (3)	−0.037 (4)	0.068 (3)	−0.023 (3)
C32	0.089 (3)	0.130 (4)	0.039 (2)	0.032 (3)	−0.010 (2)	−0.034 (2)
C33	0.0344 (14)	0.0368 (15)	0.0280 (14)	−0.0110 (11)	0.0042 (11)	−0.0094 (11)
C34	0.0395 (16)	0.0499 (18)	0.0325 (15)	0.0083 (13)	0.0013 (12)	0.0144 (13)
C35	0.054 (2)	0.071 (2)	0.0329 (17)	0.0052 (17)	0.0055 (14)	0.0095 (16)
C36	0.069 (2)	0.055 (2)	0.050 (2)	0.0221 (18)	0.0043 (18)	0.0224 (17)
C37	0.0460 (19)	0.084 (3)	0.044 (2)	0.0002 (18)	−0.0099 (15)	0.0235 (19)
C38	0.0385 (15)	0.0366 (15)	0.0312 (14)	0.0012 (12)	0.0078 (12)	−0.0053 (12)
C39	0.0534 (19)	0.0465 (18)	0.0333 (16)	0.0049 (14)	0.0116 (14)	−0.0089 (13)
C40	0.261 (8)	0.078 (3)	0.039 (2)	0.040 (4)	0.049 (4)	−0.003 (2)
C41	0.197 (7)	0.099 (4)	0.062 (3)	−0.067 (4)	0.030 (4)	−0.045 (3)
C42	0.145 (6)	0.313 (11)	0.084 (4)	0.144 (7)	−0.022 (4)	−0.105 (6)
C43	0.0424 (16)	0.0355 (15)	0.0375 (16)	−0.0018 (12)	0.0177 (13)	−0.0010 (12)
C44	0.0406 (16)	0.061 (2)	0.0201 (13)	−0.0081 (14)	0.0070 (11)	−0.0064 (13)
C45B	0.089 (6)	0.064 (4)	0.037 (3)	0.005 (4)	0.018 (3)	−0.017 (3)
C46B	0.059 (4)	0.080 (5)	0.028 (3)	−0.005 (3)	0.005 (3)	−0.006 (3)
C47B	0.065 (5)	0.159 (10)	0.044 (4)	−0.044 (6)	0.030 (4)	−0.044 (5)
C48	0.0389 (17)	0.0438 (18)	0.066 (2)	0.0078 (14)	0.0152 (16)	−0.0164 (16)
C45	0.101 (10)	0.108 (11)	0.060 (7)	−0.058 (9)	0.043 (7)	−0.044 (7)
C46	0.098 (10)	0.114 (10)	0.024 (5)	0.006 (7)	0.001 (5)	0.001 (5)
C47	0.088 (8)	0.117 (11)	0.034 (5)	0.045 (8)	0.008 (5)	−0.025 (6)
O1A	0.0248 (9)	0.0285 (9)	0.0243 (9)	0.0001 (7)	0.0036 (7)	−0.0053 (7)
O2A	0.0272 (9)	0.0242 (8)	0.0241 (9)	0.0002 (7)	0.0052 (7)	0.0053 (7)
O3A	0.0273 (9)	0.0249 (9)	0.0278 (9)	−0.0003 (7)	0.0036 (7)	−0.0031 (7)
O4A	0.0336 (9)	0.0222 (8)	0.0209 (8)	−0.0011 (7)	0.0020 (7)	−0.0011 (7)
C1A	0.0168 (11)	0.0277 (12)	0.0213 (11)	0.0036 (9)	0.0028 (9)	−0.0025 (9)
C2A	0.0222 (11)	0.0260 (12)	0.0234 (12)	0.0072 (9)	0.0057 (9)	0.0015 (10)
C3A	0.0257 (12)	0.0216 (12)	0.0274 (13)	0.0035 (9)	0.0049 (10)	0.0000 (10)
C4A	0.0230 (12)	0.0294 (13)	0.0230 (12)	0.0015 (10)	0.0023 (9)	−0.0025 (10)
C5A	0.0236 (12)	0.0343 (13)	0.0198 (12)	0.0020 (10)	0.0035 (9)	0.0047 (10)
C6A	0.0182 (11)	0.0262 (12)	0.0271 (13)	0.0027 (9)	0.0068 (9)	−0.0001 (10)
C7A	0.0228 (12)	0.0294 (13)	0.0299 (13)	−0.0029 (10)	0.0078 (10)	0.0030 (10)
C8A	0.0252 (12)	0.0181 (11)	0.0257 (12)	−0.0035 (9)	0.0050 (10)	0.0028 (9)
C9A	0.0256 (12)	0.0206 (11)	0.0296 (13)	−0.0062 (9)	0.0063 (10)	−0.0013 (10)
C10A	0.0325 (13)	0.0234 (12)	0.0263 (13)	−0.0094 (10)	0.0085 (10)	−0.0016 (10)
C11A	0.0399 (14)	0.0204 (12)	0.0228 (12)	−0.0096 (10)	0.0043 (11)	−0.0023 (9)
C12A	0.0309 (13)	0.0192 (11)	0.0295 (13)	−0.0033 (10)	0.0015 (10)	0.0017 (10)
C13A	0.0272 (12)	0.0149 (11)	0.0304 (13)	−0.0040 (9)	0.0038 (10)	0.0001 (9)
C14A	0.0278 (12)	0.0199 (11)	0.0301 (13)	0.0037 (9)	0.0075 (10)	0.0050 (10)
C15A	0.0169 (11)	0.0214 (11)	0.0248 (12)	0.0017 (9)	0.0025 (9)	0.0001 (9)
C16A	0.0182 (11)	0.0212 (11)	0.0283 (13)	0.0034 (9)	0.0051 (9)	0.0029 (9)
C17A	0.0258 (12)	0.0265 (12)	0.0247 (12)	0.0048 (10)	0.0049 (10)	0.0057 (10)
C18A	0.0218 (11)	0.0256 (12)	0.0256 (12)	0.0023 (9)	0.0042 (9)	−0.0022 (10)
C19A	0.0196 (11)	0.0220 (11)	0.0311 (13)	−0.0018 (9)	0.0056 (10)	0.0019 (10)
C20A	0.0175 (11)	0.0245 (12)	0.0261 (12)	0.0004 (9)	0.0055 (9)	0.0019 (10)
C21A	0.0225 (12)	0.0232 (12)	0.0287 (13)	−0.0022 (9)	0.0072 (10)	0.0023 (10)
C22A	0.0286 (12)	0.0164 (11)	0.0193 (11)	−0.0007 (9)	0.0027 (9)	0.0031 (9)
C23A	0.0240 (12)	0.0179 (11)	0.0244 (12)	−0.0021 (9)	0.0058 (9)	0.0074 (9)
C24A	0.0304 (13)	0.0213 (12)	0.0266 (13)	0.0020 (10)	0.0106 (10)	0.0026 (10)
C25A	0.0299 (13)	0.0264 (12)	0.0201 (12)	−0.0005 (10)	0.0047 (10)	0.0013 (10)
C26A	0.0241 (12)	0.0263 (12)	0.0244 (12)	0.0013 (10)	0.0012 (10)	0.0022 (10)
C27A	0.0284 (12)	0.0215 (11)	0.0210 (12)	0.0026 (9)	0.0063 (9)	0.0038 (9)
C28A	0.0252 (12)	0.0235 (12)	0.0259 (12)	0.0056 (9)	0.0047 (10)	0.0023 (10)
C29A	0.0314 (13)	0.0397 (15)	0.0244 (13)	0.0017 (11)	0.0027 (10)	−0.0034 (11)
C30A	0.100 (3)	0.059 (2)	0.0336 (18)	0.002 (2)	−0.0231 (19)	−0.0047 (16)
C31A	0.118 (4)	0.050 (2)	0.0384 (19)	−0.027 (2)	0.010 (2)	−0.0114 (16)
C32A	0.043 (2)	0.186 (6)	0.080 (3)	−0.002 (3)	0.015 (2)	−0.091 (4)
C33A	0.0287 (14)	0.058 (2)	0.0373 (16)	0.0015 (13)	−0.0019 (12)	−0.0110 (14)
C34A	0.0410 (15)	0.0317 (14)	0.0231 (13)	−0.0026 (11)	0.0053 (11)	0.0034 (11)
C35A	0.103 (5)	0.029 (2)	0.036 (3)	0.013 (3)	0.024 (3)	0.013 (2)
C36A	0.040 (3)	0.070 (4)	0.030 (3)	−0.007 (2)	0.000 (2)	−0.003 (2)
C37A	0.047 (3)	0.051 (3)	0.027 (2)	0.005 (2)	0.010 (2)	0.010 (2)
C38A	0.0329 (14)	0.0375 (15)	0.0268 (13)	0.0005 (11)	0.0067 (11)	0.0014 (11)
C39A	0.0415 (15)	0.0344 (14)	0.0276 (14)	−0.0011 (12)	0.0051 (12)	−0.0044 (11)
C40A	0.132 (4)	0.083 (3)	0.034 (2)	−0.039 (3)	0.016 (2)	−0.0137 (19)
C41A	0.053 (3)	0.273 (9)	0.076 (3)	−0.041 (4)	0.001 (2)	−0.099 (5)
C42A	0.275 (8)	0.048 (2)	0.042 (2)	0.037 (4)	−0.002 (3)	−0.0184 (19)
C43A	0.0440 (16)	0.0374 (15)	0.0316 (15)	0.0091 (12)	0.0081 (12)	−0.0068 (12)
C44A	0.0352 (14)	0.0373 (15)	0.0252 (13)	−0.0001 (11)	0.0036 (11)	−0.0083 (11)
C45A	0.073 (2)	0.0421 (18)	0.0461 (19)	−0.0083 (16)	0.0024 (17)	−0.0118 (15)
C46A	0.064 (2)	0.068 (2)	0.0268 (15)	−0.0078 (18)	0.0095 (15)	−0.0088 (15)
C47A	0.0448 (19)	0.100 (3)	0.0395 (19)	0.0093 (19)	−0.0080 (15)	−0.0306 (19)
C48A	0.0400 (15)	0.0280 (14)	0.0392 (16)	−0.0048 (11)	0.0074 (12)	−0.0035 (12)
C35C	0.043 (5)	0.041 (5)	0.031 (4)	−0.004 (4)	0.002 (3)	0.018 (3)
C36C	0.041 (4)	0.047 (5)	0.026 (4)	0.003 (4)	0.006 (3)	0.011 (3)
C37C	0.085 (8)	0.042 (5)	0.029 (4)	0.007 (5)	0.004 (4)	−0.004 (4)
C1G	0.091 (3)	0.092 (3)	0.056 (3)	−0.009 (3)	0.020 (2)	−0.014 (2)
Cl1G	0.1413 (12)	0.0810 (8)	0.0632 (7)	0.0040 (8)	0.0196 (7)	−0.0072 (6)
Cl2G	0.1168 (11)	0.1275 (12)	0.1013 (11)	0.0235 (9)	0.0327 (9)	−0.0333 (9)

### Geometric parameters (Å, °)

**Table:**

O1—C1	1.390 (3)	O2A—C8A	1.382 (3)
O1—C33	1.428 (3)	O2A—C38A	1.432 (3)
O2—C8	1.376 (3)	O3A—C15A	1.390 (3)
O2—C38	1.428 (3)	O3A—C43A	1.435 (3)
O3—C15	1.390 (3)	O4A—C22A	1.392 (3)
O3—C43	1.418 (3)	O4A—C48A	1.425 (3)
O4—C22	1.392 (3)	C1A—C6A	1.396 (3)
O4—C48	1.433 (3)	C1A—C2A	1.397 (3)
C1—C6	1.389 (3)	C2A—C3A	1.391 (3)
C1—C2	1.397 (3)	C2A—C28A	1.519 (3)
C2—C3	1.396 (4)	C3A—C4A	1.400 (3)
C2—C28	1.523 (3)	C3A—H3A	0.9500
C3—C4	1.397 (4)	C4A—C5A	1.397 (4)
C3—H3	0.9500	C4A—C29A	1.542 (3)
C4—C5	1.390 (4)	C5A—C6A	1.389 (3)
C4—C29	1.538 (4)	C5A—H5A	0.9500
C5—C6	1.391 (4)	C6A—C7A	1.520 (3)
C5—H5	0.9500	C7A—C9A	1.521 (3)
C6—C7	1.520 (4)	C7A—H7A1	0.9900
C7—C9	1.517 (4)	C7A—H7A2	0.9900
C7—H7A	0.9900	C8A—C9A	1.398 (3)
C7—H7B	0.9900	C8A—C13A	1.403 (3)
C8—C13	1.398 (3)	C9A—C10A	1.395 (3)
C8—C9	1.400 (4)	C10A—C11A	1.392 (4)
C9—C10	1.387 (4)	C10A—H10A	0.9500
C10—C11	1.407 (4)	C11A—C12A	1.394 (4)
C10—H10	0.9500	C11A—C34A	1.539 (3)
C11—C12	1.386 (4)	C12A—C13A	1.391 (3)
C11—C34	1.529 (4)	C12A—H12A	0.9500
C12—C13	1.398 (3)	C13A—C14A	1.515 (3)
C12—H12	0.9500	C14A—C16A	1.523 (3)
C13—C14	1.515 (4)	C14A—H14A	0.9900
C14—C16	1.527 (3)	C14A—H14B	0.9900
C14—H14C	0.9900	C15A—C16A	1.388 (3)
C14—H14D	0.9900	C15A—C20A	1.402 (3)
C15—C16	1.395 (3)	C16A—C17A	1.404 (3)
C15—C20	1.396 (3)	C17A—C18A	1.386 (3)
C16—C17	1.391 (4)	C17A—H17A	0.9500
C17—C18	1.396 (4)	C18A—C19A	1.398 (3)
C17—H17	0.9500	C18A—C39A	1.535 (4)
C18—C19	1.392 (4)	C19A—C20A	1.393 (3)
C18—C39	1.532 (4)	C19A—H19A	0.9500
C19—C20	1.387 (4)	C20A—C21A	1.516 (3)
C19—H19	0.9500	C21A—C23A	1.517 (3)
C20—C21	1.520 (3)	C21A—H21A	0.9900
C21—C23	1.517 (4)	C21A—H21B	0.9900
C21—H21C	0.9900	C22A—C27A	1.397 (3)
C21—H21D	0.9900	C22A—C23A	1.402 (3)
C22—C27	1.393 (4)	C23A—C24A	1.383 (3)
C22—C23	1.394 (3)	C24A—C25A	1.397 (4)
C23—C24	1.391 (4)	C24A—H24A	0.9500
C24—C25	1.404 (4)	C25A—C26A	1.397 (3)
C24—H24	0.9500	C25A—C44A	1.535 (3)
C25—C26	1.395 (4)	C26A—C27A	1.391 (3)
C25—C44	1.532 (4)	C26A—H26A	0.9500
C26—C27	1.391 (3)	C27A—C28A	1.524 (3)
C26—H26	0.9500	C28A—H28A	0.9900
C27—C28	1.517 (3)	C28A—H28B	0.9900
C28—H28C	0.9900	C29A—C31A	1.513 (5)
C28—H28D	0.9900	C29A—C30A	1.518 (4)
C29—C32	1.495 (5)	C29A—C32A	1.527 (5)
C29—C30	1.505 (5)	C30A—H30D	0.9800
C29—C31	1.569 (6)	C30A—H30E	0.9800
C30—H30A	0.9800	C30A—H30F	0.9800
C30—H30B	0.9800	C31A—H31D	0.9800
C30—H30C	0.9800	C31A—H31E	0.9800
C31—H31A	0.9800	C31A—H31F	0.9800
C31—H31B	0.9800	C32A—H32D	0.9800
C31—H31C	0.9800	C32A—H32E	0.9800
C32—H32A	0.9800	C32A—H32F	0.9800
C32—H32B	0.9800	C33A—H33D	0.9800
C32—H32C	0.9800	C33A—H33E	0.9800
C33—H33A	0.9800	C33A—H33F	0.9800
C33—H33B	0.9800	C34A—C37C	1.439 (9)
C33—H33C	0.9800	C34A—C35A	1.476 (5)
C34—C36	1.534 (5)	C34A—C35C	1.520 (8)
C34—C35	1.536 (5)	C34A—C36A	1.525 (5)
C34—C37	1.544 (5)	C34A—C37A	1.588 (5)
C35—H35A	0.9800	C34A—C36C	1.667 (8)
C35—H35B	0.9800	C35A—H35D	0.9800
C35—H35C	0.9800	C35A—H35E	0.9800
C36—H36A	0.9800	C35A—H35F	0.9800
C36—H36B	0.9800	C36A—H36D	0.9800
C36—H36C	0.9800	C36A—H36E	0.9800
C37—H37A	0.9800	C36A—H36F	0.9800
C37—H37B	0.9800	C37A—H37D	0.9800
C37—H37C	0.9800	C37A—H37E	0.9800
C38—H38D	0.9800	C37A—H37F	0.9800
C38—H38E	0.9800	C38A—H38A	0.9800
C38—H38F	0.9800	C38A—H38B	0.9800
C39—C42	1.488 (6)	C38A—H38C	0.9800
C39—C40	1.506 (6)	C39A—C41A	1.504 (5)
C39—C41	1.534 (6)	C39A—C40A	1.505 (5)
C40—H40A	0.9800	C39A—C42A	1.509 (5)
C40—H40B	0.9800	C40A—H40D	0.9800
C40—H40C	0.9800	C40A—H40E	0.9800
C41—H41A	0.9800	C40A—H40F	0.9800
C41—H41B	0.9800	C41A—H41D	0.9800
C41—H41C	0.9800	C41A—H41E	0.9800
C42—H42A	0.9800	C41A—H41F	0.9800
C42—H42B	0.9800	C42A—H42D	0.9800
C42—H42C	0.9800	C42A—H42E	0.9800
C43—H43A	0.9800	C42A—H42F	0.9800
C43—H43B	0.9800	C43A—H43D	0.9800
C43—H43C	0.9800	C43A—H43E	0.9800
C44—C46B	1.494 (7)	C43A—H43F	0.9800
C44—C47B	1.499 (7)	C44A—C47A	1.522 (4)
C44—C47	1.503 (11)	C44A—C45A	1.535 (4)
C44—C45	1.520 (11)	C44A—C46A	1.536 (4)
C44—C45B	1.573 (7)	C45A—H45G	0.9800
C44—C46	1.592 (12)	C45A—H45H	0.9800
C45B—H45D	0.9800	C45A—H45I	0.9800
C45B—H45E	0.9800	C46A—H46G	0.9800
C45B—H45F	0.9800	C46A—H46H	0.9800
C46B—H46D	0.9800	C46A—H46I	0.9800
C46B—H46E	0.9800	C47A—H47G	0.9800
C46B—H46F	0.9800	C47A—H47H	0.9800
C47B—H47D	0.9800	C47A—H47I	0.9800
C47B—H47E	0.9800	C48A—H48D	0.9800
C47B—H47F	0.9800	C48A—H48E	0.9800
C48—H48A	0.9800	C48A—H48F	0.9800
C48—H48B	0.9800	C35C—H35G	0.9800
C48—H48C	0.9800	C35C—H35H	0.9800
C45—H45A	0.9800	C35C—H35I	0.9800
C45—H45B	0.9800	C36C—H36G	0.9800
C45—H45C	0.9800	C36C—H36H	0.9800
C46—H46A	0.9800	C36C—H36I	0.9800
C46—H46B	0.9800	C37C—H37G	0.9800
C46—H46C	0.9800	C37C—H37H	0.9800
C47—H47A	0.9800	C37C—H37I	0.9800
C47—H47B	0.9800	C1G—Cl1G	1.732 (5)
C47—H47C	0.9800	C1G—Cl2G	1.762 (5)
O1A—C1A	1.386 (3)	C1G—H1G1	0.9900
O1A—C33A	1.433 (3)	C1G—H1G2	0.9900
			
C1—O1—C33	114.18 (18)	H47B—C47—H47C	109.5
C8—O2—C38	115.73 (19)	C1A—O1A—C33A	113.35 (19)
C15—O3—C43	113.77 (19)	C8A—O2A—C38A	115.31 (18)
C22—O4—C48	113.5 (2)	C15A—O3A—C43A	114.37 (19)
C6—C1—O1	120.0 (2)	C22A—O4A—C48A	115.01 (19)
C6—C1—C2	120.9 (2)	O1A—C1A—C6A	119.9 (2)
O1—C1—C2	119.1 (2)	O1A—C1A—C2A	119.4 (2)
C3—C2—C1	118.6 (2)	C6A—C1A—C2A	120.6 (2)
C3—C2—C28	119.6 (2)	C3A—C2A—C1A	119.0 (2)
C1—C2—C28	121.7 (2)	C3A—C2A—C28A	120.4 (2)
C2—C3—C4	121.9 (2)	C1A—C2A—C28A	120.5 (2)
C2—C3—H3	119.0	C2A—C3A—C4A	121.8 (2)
C4—C3—H3	119.0	C2A—C3A—H3A	119.1
C5—C4—C3	117.3 (2)	C4A—C3A—H3A	119.1
C5—C4—C29	120.7 (2)	C5A—C4A—C3A	117.4 (2)
C3—C4—C29	122.0 (2)	C5A—C4A—C29A	120.9 (2)
C4—C5—C6	122.5 (2)	C3A—C4A—C29A	121.7 (2)
C4—C5—H5	118.7	C6A—C5A—C4A	122.3 (2)
C6—C5—H5	118.7	C6A—C5A—H5A	118.8
C1—C6—C5	118.6 (2)	C4A—C5A—H5A	118.8
C1—C6—C7	121.4 (2)	C5A—C6A—C1A	118.7 (2)
C5—C6—C7	119.9 (2)	C5A—C6A—C7A	120.3 (2)
C9—C7—C6	110.1 (2)	C1A—C6A—C7A	121.0 (2)
C9—C7—H7A	109.6	C6A—C7A—C9A	112.4 (2)
C6—C7—H7A	109.6	C6A—C7A—H7A1	109.1
C9—C7—H7B	109.6	C9A—C7A—H7A1	109.1
C6—C7—H7B	109.6	C6A—C7A—H7A2	109.1
H7A—C7—H7B	108.2	C9A—C7A—H7A2	109.1
O2—C8—C13	119.4 (2)	H7A1—C7A—H7A2	107.9
O2—C8—C9	119.4 (2)	O2A—C8A—C9A	119.6 (2)
C13—C8—C9	121.0 (2)	O2A—C8A—C13A	119.1 (2)
C10—C9—C8	118.4 (2)	C9A—C8A—C13A	121.1 (2)
C10—C9—C7	121.5 (2)	C10A—C9A—C8A	118.3 (2)
C8—C9—C7	119.9 (2)	C10A—C9A—C7A	121.2 (2)
C9—C10—C11	122.5 (2)	C8A—C9A—C7A	120.4 (2)
C9—C10—H10	118.8	C11A—C10A—C9A	122.5 (2)
C11—C10—H10	118.8	C11A—C10A—H10A	118.8
C12—C11—C10	117.2 (2)	C9A—C10A—H10A	118.8
C12—C11—C34	123.2 (2)	C10A—C11A—C12A	117.4 (2)
C10—C11—C34	119.5 (2)	C10A—C11A—C34A	121.8 (2)
C11—C12—C13	122.4 (2)	C12A—C11A—C34A	120.8 (2)
C11—C12—H12	118.8	C13A—C12A—C11A	122.6 (2)
C13—C12—H12	118.8	C13A—C12A—H12A	118.7
C8—C13—C12	118.6 (2)	C11A—C12A—H12A	118.7
C8—C13—C14	120.2 (2)	C12A—C13A—C8A	118.2 (2)
C12—C13—C14	121.3 (2)	C12A—C13A—C14A	121.6 (2)
C13—C14—C16	111.7 (2)	C8A—C13A—C14A	120.0 (2)
C13—C14—H14C	109.3	C13A—C14A—C16A	111.04 (19)
C16—C14—H14C	109.3	C13A—C14A—H14A	109.4
C13—C14—H14D	109.3	C16A—C14A—H14A	109.4
C16—C14—H14D	109.3	C13A—C14A—H14B	109.4
H14C—C14—H14D	107.9	C16A—C14A—H14B	109.4
O3—C15—C16	120.0 (2)	H14A—C14A—H14B	108.0
O3—C15—C20	119.4 (2)	C16A—C15A—O3A	119.8 (2)
C16—C15—C20	120.5 (2)	C16A—C15A—C20A	120.7 (2)
C17—C16—C15	119.1 (2)	O3A—C15A—C20A	119.2 (2)
C17—C16—C14	119.7 (2)	C15A—C16A—C17A	118.8 (2)
C15—C16—C14	121.1 (2)	C15A—C16A—C14A	121.8 (2)
C16—C17—C18	121.8 (2)	C17A—C16A—C14A	119.1 (2)
C16—C17—H17	119.1	C18A—C17A—C16A	122.2 (2)
C18—C17—H17	119.1	C18A—C17A—H17A	118.9
C19—C18—C17	117.3 (2)	C16A—C17A—H17A	118.9
C19—C18—C39	121.3 (2)	C17A—C18A—C19A	117.2 (2)
C17—C18—C39	121.5 (2)	C17A—C18A—C39A	122.3 (2)
C20—C19—C18	122.6 (2)	C19A—C18A—C39A	120.5 (2)
C20—C19—H19	118.7	C20A—C19A—C18A	122.6 (2)
C18—C19—H19	118.7	C20A—C19A—H19A	118.7
C19—C20—C15	118.6 (2)	C18A—C19A—H19A	118.7
C19—C20—C21	120.9 (2)	C19A—C20A—C15A	118.3 (2)
C15—C20—C21	120.3 (2)	C19A—C20A—C21A	120.4 (2)
C23—C21—C20	113.2 (2)	C15A—C20A—C21A	121.2 (2)
C23—C21—H21C	108.9	C20A—C21A—C23A	113.02 (19)
C20—C21—H21C	108.9	C20A—C21A—H21A	109.0
C23—C21—H21D	108.9	C23A—C21A—H21A	109.0
C20—C21—H21D	108.9	C20A—C21A—H21B	109.0
H21C—C21—H21D	107.7	C23A—C21A—H21B	109.0
O4—C22—C27	119.0 (2)	H21A—C21A—H21B	107.8
O4—C22—C23	119.3 (2)	O4A—C22A—C27A	120.0 (2)
C27—C22—C23	121.6 (2)	O4A—C22A—C23A	119.0 (2)
C24—C23—C22	118.3 (2)	C27A—C22A—C23A	120.9 (2)
C24—C23—C21	120.5 (2)	C24A—C23A—C22A	118.4 (2)
C22—C23—C21	121.1 (2)	C24A—C23A—C21A	121.2 (2)
C23—C24—C25	122.3 (2)	C22A—C23A—C21A	120.4 (2)
C23—C24—H24	118.8	C23A—C24A—C25A	122.8 (2)
C25—C24—H24	118.8	C23A—C24A—H24A	118.6
C26—C25—C24	117.0 (2)	C25A—C24A—H24A	118.6
C26—C25—C44	121.1 (2)	C26A—C25A—C24A	117.1 (2)
C24—C25—C44	121.9 (2)	C26A—C25A—C44A	122.6 (2)
C27—C26—C25	122.6 (2)	C24A—C25A—C44A	120.3 (2)
C27—C26—H26	118.7	C27A—C26A—C25A	122.2 (2)
C25—C26—H26	118.7	C27A—C26A—H26A	118.9
C26—C27—C22	118.2 (2)	C25A—C26A—H26A	118.9
C26—C27—C28	120.6 (2)	C26A—C27A—C22A	118.6 (2)
C22—C27—C28	121.1 (2)	C26A—C27A—C28A	120.6 (2)
C27—C28—C2	113.43 (19)	C22A—C27A—C28A	120.7 (2)
C27—C28—H28C	108.9	C2A—C28A—C27A	113.01 (19)
C2—C28—H28C	108.9	C2A—C28A—H28A	109.0
C27—C28—H28D	108.9	C27A—C28A—H28A	109.0
C2—C28—H28D	108.9	C2A—C28A—H28B	109.0
H28C—C28—H28D	107.7	C27A—C28A—H28B	109.0
C32—C29—C30	112.5 (4)	H28A—C28A—H28B	107.8
C32—C29—C4	111.7 (3)	C31A—C29A—C30A	107.1 (3)
C30—C29—C4	112.7 (3)	C31A—C29A—C32A	109.0 (4)
C32—C29—C31	105.4 (4)	C30A—C29A—C32A	108.6 (3)
C30—C29—C31	106.8 (4)	C31A—C29A—C4A	112.7 (2)
C4—C29—C31	107.2 (3)	C30A—C29A—C4A	111.1 (2)
C29—C30—H30A	109.5	C32A—C29A—C4A	108.3 (2)
C29—C30—H30B	109.5	C29A—C30A—H30D	109.5
H30A—C30—H30B	109.5	C29A—C30A—H30E	109.5
C29—C30—H30C	109.5	H30D—C30A—H30E	109.5
H30A—C30—H30C	109.5	C29A—C30A—H30F	109.5
H30B—C30—H30C	109.5	H30D—C30A—H30F	109.5
C29—C31—H31A	109.5	H30E—C30A—H30F	109.5
C29—C31—H31B	109.5	C29A—C31A—H31D	109.5
H31A—C31—H31B	109.5	C29A—C31A—H31E	109.5
C29—C31—H31C	109.5	H31D—C31A—H31E	109.5
H31A—C31—H31C	109.5	C29A—C31A—H31F	109.5
H31B—C31—H31C	109.5	H31D—C31A—H31F	109.5
C29—C32—H32A	109.5	H31E—C31A—H31F	109.5
C29—C32—H32B	109.5	C29A—C32A—H32D	109.5
H32A—C32—H32B	109.5	C29A—C32A—H32E	109.5
C29—C32—H32C	109.5	H32D—C32A—H32E	109.5
H32A—C32—H32C	109.5	C29A—C32A—H32F	109.5
H32B—C32—H32C	109.5	H32D—C32A—H32F	109.5
O1—C33—H33A	109.5	H32E—C32A—H32F	109.5
O1—C33—H33B	109.5	O1A—C33A—H33D	109.5
H33A—C33—H33B	109.5	O1A—C33A—H33E	109.5
O1—C33—H33C	109.5	H33D—C33A—H33E	109.5
H33A—C33—H33C	109.5	O1A—C33A—H33F	109.5
H33B—C33—H33C	109.5	H33D—C33A—H33F	109.5
C11—C34—C36	109.7 (3)	H33E—C33A—H33F	109.5
C11—C34—C35	109.2 (2)	C37C—C34A—C35A	138.4 (5)
C36—C34—C35	110.2 (3)	C37C—C34A—C35C	115.6 (6)
C11—C34—C37	112.3 (3)	C35A—C34A—C35C	48.3 (4)
C36—C34—C37	108.5 (3)	C37C—C34A—C36A	52.3 (5)
C35—C34—C37	107.0 (3)	C35A—C34A—C36A	113.3 (4)
C34—C35—H35A	109.5	C35C—C34A—C36A	143.0 (4)
C34—C35—H35B	109.5	C37C—C34A—C11A	111.1 (4)
H35A—C35—H35B	109.5	C35A—C34A—C11A	110.6 (3)
C34—C35—H35C	109.5	C35C—C34A—C11A	109.1 (3)
H35A—C35—H35C	109.5	C36A—C34A—C11A	107.7 (3)
H35B—C35—H35C	109.5	C37C—C34A—C37A	56.3 (5)
C34—C36—H36A	109.5	C35A—C34A—C37A	107.3 (4)
C34—C36—H36B	109.5	C35C—C34A—C37A	63.1 (4)
H36A—C36—H36B	109.5	C36A—C34A—C37A	106.5 (3)
C34—C36—H36C	109.5	C11A—C34A—C37A	111.5 (3)
H36A—C36—H36C	109.5	C37C—C34A—C36C	106.3 (5)
H36B—C36—H36C	109.5	C35A—C34A—C36C	56.8 (4)
C34—C37—H37A	109.5	C35C—C34A—C36C	102.6 (5)
C34—C37—H37B	109.5	C36A—C34A—C36C	59.0 (4)
H37A—C37—H37B	109.5	C11A—C34A—C36C	111.9 (3)
C34—C37—H37C	109.5	C37A—C34A—C36C	136.7 (4)
H37A—C37—H37C	109.5	C34A—C35A—H35D	109.5
H37B—C37—H37C	109.5	C34A—C35A—H35E	109.5
O2—C38—H38D	109.5	C34A—C35A—H35F	109.5
O2—C38—H38E	109.5	C34A—C36A—H36D	109.5
H38D—C38—H38E	109.5	C34A—C36A—H36E	109.5
O2—C38—H38F	109.5	C34A—C36A—H36F	109.5
H38D—C38—H38F	109.5	C34A—C37A—H37D	109.5
H38E—C38—H38F	109.5	C34A—C37A—H37E	109.5
C42—C39—C40	112.0 (5)	C34A—C37A—H37F	109.5
C42—C39—C18	110.8 (3)	O2A—C38A—H38A	109.5
C40—C39—C18	112.3 (3)	O2A—C38A—H38B	109.5
C42—C39—C41	104.2 (6)	H38A—C38A—H38B	109.5
C40—C39—C41	109.1 (4)	O2A—C38A—H38C	109.5
C18—C39—C41	108.0 (3)	H38A—C38A—H38C	109.5
C39—C40—H40A	109.5	H38B—C38A—H38C	109.5
C39—C40—H40B	109.5	C41A—C39A—C40A	110.0 (4)
H40A—C40—H40B	109.5	C41A—C39A—C42A	107.0 (5)
C39—C40—H40C	109.5	C40A—C39A—C42A	107.8 (4)
H40A—C40—H40C	109.5	C41A—C39A—C18A	109.6 (3)
H40B—C40—H40C	109.5	C40A—C39A—C18A	112.0 (3)
C39—C41—H41A	109.5	C42A—C39A—C18A	110.3 (3)
C39—C41—H41B	109.5	C39A—C40A—H40D	109.5
H41A—C41—H41B	109.5	C39A—C40A—H40E	109.5
C39—C41—H41C	109.5	H40D—C40A—H40E	109.5
H41A—C41—H41C	109.5	C39A—C40A—H40F	109.5
H41B—C41—H41C	109.5	H40D—C40A—H40F	109.5
C39—C42—H42A	109.5	H40E—C40A—H40F	109.5
C39—C42—H42B	109.5	C39A—C41A—H41D	109.5
H42A—C42—H42B	109.5	C39A—C41A—H41E	109.5
C39—C42—H42C	109.5	H41D—C41A—H41E	109.5
H42A—C42—H42C	109.5	C39A—C41A—H41F	109.5
H42B—C42—H42C	109.5	H41D—C41A—H41F	109.5
O3—C43—H43A	109.5	H41E—C41A—H41F	109.5
O3—C43—H43B	109.5	C39A—C42A—H42D	109.5
H43A—C43—H43B	109.5	C39A—C42A—H42E	109.5
O3—C43—H43C	109.5	H42D—C42A—H42E	109.5
H43A—C43—H43C	109.5	C39A—C42A—H42F	109.5
H43B—C43—H43C	109.5	H42D—C42A—H42F	109.5
C46B—C44—C47B	110.4 (5)	H42E—C42A—H42F	109.5
C46B—C44—C47	141.9 (5)	O3A—C43A—H43D	109.5
C47B—C44—C47	44.2 (6)	O3A—C43A—H43E	109.5
C46B—C44—C45	57.4 (7)	H43D—C43A—H43E	109.5
C47B—C44—C45	131.4 (5)	O3A—C43A—H43F	109.5
C47—C44—C45	113.3 (9)	H43D—C43A—H43F	109.5
C46B—C44—C25	107.5 (3)	H43E—C43A—H43F	109.5
C47B—C44—C25	113.5 (3)	C47A—C44A—C25A	112.6 (2)
C47—C44—C25	109.2 (4)	C47A—C44A—C45A	108.9 (3)
C45—C44—C25	114.9 (4)	C25A—C44A—C45A	108.7 (2)
C46B—C44—C45B	108.1 (5)	C47A—C44A—C46A	108.2 (3)
C47B—C44—C45B	108.3 (6)	C25A—C44A—C46A	110.1 (2)
C47—C44—C45B	68.6 (7)	C45A—C44A—C46A	108.2 (3)
C45—C44—C45B	51.3 (6)	C44A—C45A—H45G	109.5
C25—C44—C45B	108.9 (3)	C44A—C45A—H45H	109.5
C46B—C44—C46	49.5 (5)	H45G—C45A—H45H	109.5
C47B—C44—C46	65.7 (7)	C44A—C45A—H45I	109.5
C47—C44—C46	108.9 (8)	H45G—C45A—H45I	109.5
C45—C44—C46	102.9 (8)	H45H—C45A—H45I	109.5
C25—C44—C46	107.2 (5)	C44A—C46A—H46G	109.5
C45B—C44—C46	142.3 (5)	C44A—C46A—H46H	109.5
C44—C45B—H45D	109.5	H46G—C46A—H46H	109.5
C44—C45B—H45E	109.5	C44A—C46A—H46I	109.5
C44—C45B—H45F	109.5	H46G—C46A—H46I	109.5
C44—C46B—H46D	109.5	H46H—C46A—H46I	109.5
C44—C46B—H46E	109.5	C44A—C47A—H47G	109.5
C44—C46B—H46F	109.5	C44A—C47A—H47H	109.5
C44—C47B—H47D	109.5	H47G—C47A—H47H	109.5
C44—C47B—H47E	109.5	C44A—C47A—H47I	109.5
C44—C47B—H47F	109.5	H47G—C47A—H47I	109.5
O4—C48—H48A	109.5	H47H—C47A—H47I	109.5
O4—C48—H48B	109.5	O4A—C48A—H48D	109.5
H48A—C48—H48B	109.5	O4A—C48A—H48E	109.5
O4—C48—H48C	109.5	H48D—C48A—H48E	109.5
H48A—C48—H48C	109.5	O4A—C48A—H48F	109.5
H48B—C48—H48C	109.5	H48D—C48A—H48F	109.5
C44—C45—H45A	109.5	H48E—C48A—H48F	109.5
C44—C45—H45B	109.5	C34A—C35C—H35G	109.5
H45A—C45—H45B	109.5	C34A—C35C—H35H	109.5
C44—C45—H45C	109.5	C34A—C35C—H35I	109.5
H45A—C45—H45C	109.5	C34A—C36C—H36G	109.5
H45B—C45—H45C	109.5	C34A—C36C—H36H	109.5
C44—C46—H46A	109.5	C34A—C36C—H36I	109.5
C44—C46—H46B	109.5	C34A—C37C—H37G	109.5
H46A—C46—H46B	109.5	C34A—C37C—H37H	109.5
C44—C46—H46C	109.5	C34A—C37C—H37I	109.5
H46A—C46—H46C	109.5	Cl1G—C1G—Cl2G	112.1 (2)
H46B—C46—H46C	109.5	Cl1G—C1G—H1G1	109.2
C44—C47—H47A	109.5	Cl2G—C1G—H1G1	109.2
C44—C47—H47B	109.5	Cl1G—C1G—H1G2	109.2
H47A—C47—H47B	109.5	Cl2G—C1G—H1G2	109.2
C44—C47—H47C	109.5	H1G1—C1G—H1G2	107.9
H47A—C47—H47C	109.5		
			
C33—O1—C1—C6	−94.5 (3)	C33A—O1A—C1A—C6A	−98.2 (3)
C33—O1—C1—C2	89.2 (3)	C33A—O1A—C1A—C2A	84.6 (3)
C6—C1—C2—C3	2.9 (3)	O1A—C1A—C2A—C3A	−178.6 (2)
O1—C1—C2—C3	179.2 (2)	C6A—C1A—C2A—C3A	4.1 (3)
C6—C1—C2—C28	−174.1 (2)	O1A—C1A—C2A—C28A	4.3 (3)
O1—C1—C2—C28	2.2 (3)	C6A—C1A—C2A—C28A	−172.9 (2)
C1—C2—C3—C4	0.3 (4)	C1A—C2A—C3A—C4A	−2.2 (3)
C28—C2—C3—C4	177.4 (2)	C28A—C2A—C3A—C4A	174.8 (2)
C2—C3—C4—C5	−2.3 (4)	C2A—C3A—C4A—C5A	−0.5 (3)
C2—C3—C4—C29	177.4 (3)	C2A—C3A—C4A—C29A	177.5 (2)
C3—C4—C5—C6	1.2 (4)	C3A—C4A—C5A—C6A	1.4 (3)
C29—C4—C5—C6	−178.6 (3)	C29A—C4A—C5A—C6A	−176.7 (2)
O1—C1—C6—C5	179.8 (2)	C4A—C5A—C6A—C1A	0.5 (3)
C2—C1—C6—C5	−4.0 (3)	C4A—C5A—C6A—C7A	−177.8 (2)
O1—C1—C6—C7	−4.3 (3)	O1A—C1A—C6A—C5A	179.5 (2)
C2—C1—C6—C7	172.0 (2)	C2A—C1A—C6A—C5A	−3.3 (3)
C4—C5—C6—C1	1.9 (4)	O1A—C1A—C6A—C7A	−2.2 (3)
C4—C5—C6—C7	−174.1 (2)	C2A—C1A—C6A—C7A	175.0 (2)
C1—C6—C7—C9	−113.2 (3)	C5A—C6A—C7A—C9A	66.0 (3)
C5—C6—C7—C9	62.7 (3)	C1A—C6A—C7A—C9A	−112.3 (2)
C38—O2—C8—C13	−95.4 (3)	C38A—O2A—C8A—C9A	93.6 (3)
C38—O2—C8—C9	89.5 (3)	C38A—O2A—C8A—C13A	−91.3 (3)
O2—C8—C9—C10	175.6 (2)	O2A—C8A—C9A—C10A	175.4 (2)
C13—C8—C9—C10	0.7 (4)	C13A—C8A—C9A—C10A	0.4 (3)
O2—C8—C9—C7	0.8 (3)	O2A—C8A—C9A—C7A	−1.9 (3)
C13—C8—C9—C7	−174.2 (2)	C13A—C8A—C9A—C7A	−176.9 (2)
C6—C7—C9—C10	−106.5 (3)	C6A—C7A—C9A—C10A	−110.0 (3)
C6—C7—C9—C8	68.2 (3)	C6A—C7A—C9A—C8A	67.2 (3)
C8—C9—C10—C11	−1.7 (4)	C8A—C9A—C10A—C11A	−1.0 (4)
C7—C9—C10—C11	173.1 (2)	C7A—C9A—C10A—C11A	176.2 (2)
C9—C10—C11—C12	1.8 (4)	C9A—C10A—C11A—C12A	1.1 (4)
C9—C10—C11—C34	−177.0 (2)	C9A—C10A—C11A—C34A	−177.6 (2)
C10—C11—C12—C13	−0.9 (4)	C10A—C11A—C12A—C13A	−0.6 (4)
C34—C11—C12—C13	177.8 (2)	C34A—C11A—C12A—C13A	178.1 (2)
O2—C8—C13—C12	−174.8 (2)	C11A—C12A—C13A—C8A	0.0 (3)
C9—C8—C13—C12	0.2 (3)	C11A—C12A—C13A—C14A	−175.7 (2)
O2—C8—C13—C14	4.4 (3)	O2A—C8A—C13A—C12A	−174.9 (2)
C9—C8—C13—C14	179.4 (2)	C9A—C8A—C13A—C12A	0.1 (3)
C11—C12—C13—C8	0.0 (4)	O2A—C8A—C13A—C14A	0.9 (3)
C11—C12—C13—C14	−179.2 (2)	C9A—C8A—C13A—C14A	175.9 (2)
C8—C13—C14—C16	−70.2 (3)	C12A—C13A—C14A—C16A	109.1 (2)
C12—C13—C14—C16	109.0 (3)	C8A—C13A—C14A—C16A	−66.5 (3)
C43—O3—C15—C16	90.7 (3)	C43A—O3A—C15A—C16A	102.0 (3)
C43—O3—C15—C20	−92.6 (3)	C43A—O3A—C15A—C20A	−83.2 (3)
O3—C15—C16—C17	179.2 (2)	O3A—C15A—C16A—C17A	178.2 (2)
C20—C15—C16—C17	2.5 (3)	C20A—C15A—C16A—C17A	3.5 (3)
O3—C15—C16—C14	1.9 (3)	O3A—C15A—C16A—C14A	3.6 (3)
C20—C15—C16—C14	−174.8 (2)	C20A—C15A—C16A—C14A	−171.1 (2)
C13—C14—C16—C17	−65.4 (3)	C13A—C14A—C16A—C15A	114.8 (2)
C13—C14—C16—C15	112.0 (3)	C13A—C14A—C16A—C17A	−59.7 (3)
C15—C16—C17—C18	0.1 (4)	C15A—C16A—C17A—C18A	−1.3 (3)
C14—C16—C17—C18	177.5 (2)	C14A—C16A—C17A—C18A	173.4 (2)
C16—C17—C18—C19	−1.6 (4)	C16A—C17A—C18A—C19A	−0.6 (3)
C16—C17—C18—C39	179.3 (2)	C16A—C17A—C18A—C39A	−179.2 (2)
C17—C18—C19—C20	0.6 (4)	C17A—C18A—C19A—C20A	0.3 (3)
C39—C18—C19—C20	179.6 (2)	C39A—C18A—C19A—C20A	178.9 (2)
C18—C19—C20—C15	2.0 (4)	C18A—C19A—C20A—C15A	1.9 (3)
C18—C19—C20—C21	−174.7 (2)	C18A—C19A—C20A—C21A	−173.6 (2)
O3—C15—C20—C19	179.8 (2)	C16A—C15A—C20A—C19A	−3.8 (3)
C16—C15—C20—C19	−3.5 (3)	O3A—C15A—C20A—C19A	−178.47 (19)
O3—C15—C20—C21	−3.6 (3)	C16A—C15A—C20A—C21A	171.6 (2)
C16—C15—C20—C21	173.2 (2)	O3A—C15A—C20A—C21A	−3.1 (3)
C19—C20—C21—C23	116.1 (3)	C19A—C20A—C21A—C23A	114.0 (2)
C15—C20—C21—C23	−60.4 (3)	C15A—C20A—C21A—C23A	−61.3 (3)
C48—O4—C22—C27	95.8 (3)	C48A—O4A—C22A—C27A	87.2 (3)
C48—O4—C22—C23	−87.3 (3)	C48A—O4A—C22A—C23A	−96.8 (2)
O4—C22—C23—C24	−178.9 (2)	O4A—C22A—C23A—C24A	−177.34 (19)
C27—C22—C23—C24	−2.1 (3)	C27A—C22A—C23A—C24A	−1.4 (3)
O4—C22—C23—C21	−1.9 (3)	O4A—C22A—C23A—C21A	1.0 (3)
C27—C22—C23—C21	174.9 (2)	C27A—C22A—C23A—C21A	177.0 (2)
C20—C21—C23—C24	115.1 (3)	C20A—C21A—C23A—C24A	113.6 (2)
C20—C21—C23—C22	−61.7 (3)	C20A—C21A—C23A—C22A	−64.7 (3)
C22—C23—C24—C25	0.5 (4)	C22A—C23A—C24A—C25A	1.4 (3)
C21—C23—C24—C25	−176.4 (2)	C21A—C23A—C24A—C25A	−177.0 (2)
C23—C24—C25—C26	1.0 (4)	C23A—C24A—C25A—C26A	−1.2 (4)
C23—C24—C25—C44	179.0 (2)	C23A—C24A—C25A—C44A	176.9 (2)
C24—C25—C26—C27	−1.2 (4)	C24A—C25A—C26A—C27A	0.9 (4)
C44—C25—C26—C27	−179.2 (2)	C44A—C25A—C26A—C27A	−177.1 (2)
C25—C26—C27—C22	−0.2 (4)	C25A—C26A—C27A—C22A	−0.9 (4)
C25—C26—C27—C28	176.3 (2)	C25A—C26A—C27A—C28A	176.8 (2)
O4—C22—C27—C26	178.8 (2)	O4A—C22A—C27A—C26A	177.1 (2)
C23—C22—C27—C26	1.9 (3)	C23A—C22A—C27A—C26A	1.1 (3)
O4—C22—C27—C28	2.2 (3)	O4A—C22A—C27A—C28A	−0.6 (3)
C23—C22—C27—C28	−174.6 (2)	C23A—C22A—C27A—C28A	−176.5 (2)
C26—C27—C28—C2	−109.2 (3)	C3A—C2A—C28A—C27A	−118.3 (2)
C22—C27—C28—C2	67.3 (3)	C1A—C2A—C28A—C27A	58.7 (3)
C3—C2—C28—C27	−118.8 (3)	C26A—C27A—C28A—C2A	−115.3 (2)
C1—C2—C28—C27	58.2 (3)	C22A—C27A—C28A—C2A	62.4 (3)
C5—C4—C29—C32	−41.0 (5)	C5A—C4A—C29A—C31A	−163.8 (3)
C3—C4—C29—C32	139.2 (4)	C3A—C4A—C29A—C31A	18.3 (4)
C5—C4—C29—C30	−168.9 (4)	C5A—C4A—C29A—C30A	−43.6 (4)
C3—C4—C29—C30	11.4 (5)	C3A—C4A—C29A—C30A	138.4 (3)
C5—C4—C29—C31	73.9 (4)	C5A—C4A—C29A—C32A	75.5 (4)
C3—C4—C29—C31	−105.8 (4)	C3A—C4A—C29A—C32A	−102.4 (4)
C12—C11—C34—C36	118.2 (3)	C10A—C11A—C34A—C37C	60.6 (6)
C10—C11—C34—C36	−63.1 (3)	C12A—C11A—C34A—C37C	−118.1 (6)
C12—C11—C34—C35	−121.0 (3)	C10A—C11A—C34A—C35A	−119.5 (4)
C10—C11—C34—C35	57.7 (4)	C12A—C11A—C34A—C35A	61.8 (4)
C12—C11—C34—C37	−2.5 (4)	C10A—C11A—C34A—C35C	−68.0 (5)
C10—C11—C34—C37	176.2 (3)	C12A—C11A—C34A—C35C	113.3 (5)
C19—C18—C39—C42	−36.7 (6)	C10A—C11A—C34A—C36A	116.2 (3)
C17—C18—C39—C42	142.4 (5)	C12A—C11A—C34A—C36A	−62.5 (4)
C19—C18—C39—C40	−162.8 (4)	C10A—C11A—C34A—C37A	−0.3 (4)
C17—C18—C39—C40	16.3 (5)	C12A—C11A—C34A—C37A	−178.9 (3)
C19—C18—C39—C41	76.9 (4)	C10A—C11A—C34A—C36C	179.1 (4)
C17—C18—C39—C41	−104.1 (4)	C12A—C11A—C34A—C36C	0.5 (5)
C26—C25—C44—C46B	−64.9 (4)	C17A—C18A—C39A—C41A	−103.5 (4)
C24—C25—C44—C46B	117.2 (4)	C19A—C18A—C39A—C41A	77.9 (5)
C26—C25—C44—C47B	172.7 (6)	C17A—C18A—C39A—C40A	18.9 (4)
C24—C25—C44—C47B	−5.2 (7)	C19A—C18A—C39A—C40A	−159.7 (3)
C26—C25—C44—C47	125.2 (8)	C17A—C18A—C39A—C42A	139.0 (4)
C24—C25—C44—C47	−52.7 (8)	C19A—C18A—C39A—C42A	−39.6 (5)
C26—C25—C44—C45	−3.3 (8)	C26A—C25A—C44A—C47A	−7.4 (4)
C24—C25—C44—C45	178.8 (8)	C24A—C25A—C44A—C47A	174.6 (3)
C26—C25—C44—C45B	52.0 (5)	C26A—C25A—C44A—C45A	113.3 (3)
C24—C25—C44—C45B	−125.9 (4)	C24A—C25A—C44A—C45A	−64.6 (3)
C26—C25—C44—C46	−116.9 (6)	C26A—C25A—C44A—C46A	−128.4 (3)
C24—C25—C44—C46	65.2 (6)	C24A—C25A—C44A—C46A	53.7 (3)

### Hydrogen-bond geometry (Å, °)

**Table:**

*D*—H···*A*	*D*—H	H···*A*	*D*···*A*	*D*—H···*A*
C33A—H33E···Cl1G^i^	0.98	2.92	3.711 (3)	139
C46—H46A···Cl2G^ii^	0.98	2.89	3.551 (10)	126
C7A—H7A1···CgC^i^	0.99	2.88	3.733 (2)	145
C33—H33A···CgA'^iii^	0.98	2.74	3.549 (3)	140
C48—H48A···CgC'	0.98	2.76	3.526 (3)	136

Symmetry codes: (i) *x*+1, *y*, *z*; (ii) −*x*+1, −*y*+1, −*z*+2; (iii) −*x*+2, *y*+1/2, −*z*+3/2.
